# Experimental Study on the Blast Resistance Performance of FRP Grid & Mortar Reinforced Concrete Arch Structure

**DOI:** 10.3390/ma15207149

**Published:** 2022-10-14

**Authors:** Meirong Jiang, Shihu Qi, Shikun Pu, Peng Wang, Bo Wang, Zhanzhan Du

**Affiliations:** 1 School of Architecture Engineering, Nanjing Institute of Technology, Nanjing 211176, China; 2 Nanjing Urban Construction Management Group Co., Ltd., Nanjing 210006, China; 3 State Key Laboratory for Disaster Prevention & Mitigation of Explosion & Impact, PLA University of Science and Technology, Nanjing 210007, China

**Keywords:** FRP grid & mortar, concrete arch, blast resistance, experimental study

## Abstract

In order to verify the feasibility of using FRP grid and mortar reinforcement technology to enhance the blast resistance of concrete arch structures, this paper designed and fabricated FRP grid and mortar reinforced concrete arch structures and conducted blast resistance tests in the field. A detailed design of anti-explosion scheme was carried out before the experiment. The tests were conducted to observe the structural cracking, concrete collapse, and reinforcement peeling of FRP grid and mortar reinforced concrete arch under the explosion. In order to compare the anti-explosion performance with the protective arch structures in other literature, the explosion of 2 kg TNT with a blast distance of 600 mm was selected. After the explosion, it was found that the blast resistance of the FRP grid and mortar reinforced concrete arch was significantly higher than that of the unreinforced arch, and the concrete arch reinforced with FRP grid and mortar has a better damage patterns and improved blast resistance performance than that of the FRP and steel plate reinforced arch. According to the research results, the FRP grid and mortar composite reinforcement technology can be used to enhance the blast resistance of arch structures in protection projects.

## 1. Introduction

As a special form of structure, the arch structure has more excellent force performance than other structural members, such as beams and slabs. This is mainly because it can transform the vertical load into horizontal thrust at the foot of the arch, thus reducing the bending moment and shear force caused by the load. This advantage is why the arch structure often used in the construction of various practical projects, such as the top of the tunnel lining and the bridge cavity underneath the bridge. Likewise, arch structures have many applications in the field of defense engineering, such as aircraft caverns.

The arch structure as protective engineering may be subject to dynamic loads, such as blast impacts and earthquakes, and may lose its load-bearing capacity [1,2]. For the old concrete arch structure with damage situation, in order to improve the arch structure in peacetime bearing capacity as well as wartime protection capacity, it is more economical, convenient, and fast to strengthen and renovate it compared with demolition and new construction. In order to enhance the old concrete arch structure’s engineering resistance and service life, it is very necessary to carry out scientific and reasonable reinforcement of the arch.

**Figure 1:**
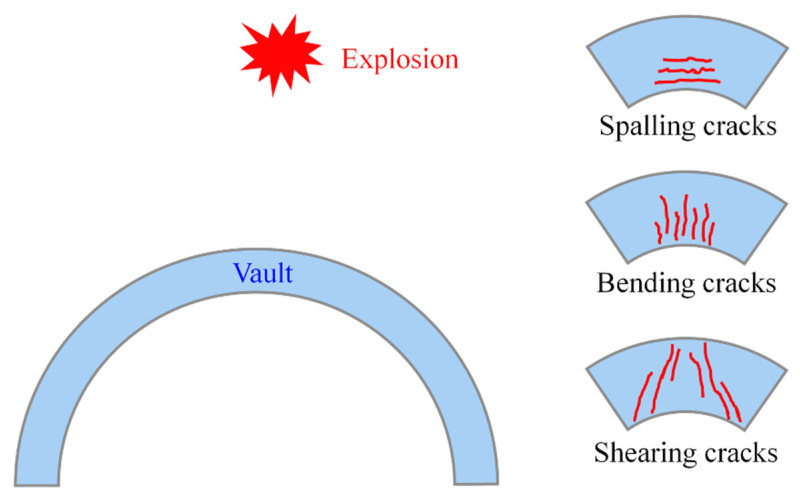
Common damages of RC arch structures subjected to the explosion [5].

In recent years, the development of science and technology has led to the emergence of various new advanced weapons, which put forward higher requirements for the resistance of protective engineering. Many underground projects built according to the original technology have not been able to give full play to the role of protection. Concrete structural members subjected to blast load behave differently compared to quasi-static loaded members [3,4]. Concrete members may experience large shear forces, bending moments, stress waves that may cause concrete cracking, and concrete spalling. Moreover, under the explosion above the RC arch, the most damaged part of the arch structure is the vault, as shown in Figure 1. Meanwhile, the stress wave caused by the explosion will induce concrete spalling at the opposite side from the loaded one [5]. Therefore, it is of great importance to use new materials and structural forms with better mechanical properties to strengthen and retrofit protective arch structures with higher blast resistance.

Fiber-reinforced composites (FRP) can be used to strengthen RC arches. Researchers found that FRP strengthening plays an important blast protective role in the concrete arch. Indeed, its effects in controlling the shearing crack and spalling, decreasing the dynamic deformation of the arch structure, and improving the structural stability are remarkable [6]. FRPs have been widely used in various fields of civil engineering [7,8]. It is important to predict the strain of FRP material and compressive strength of concrete, this is directly related to whether the reinforcement material can exert the best reinforcement effect. However, the particularity of the force form of FRP-confined concrete structures increases the difficulty of prediction. Abdalrhman [9] explored the robustness of three well-established ensemble machine learning models in the field of computer science to design FRP composite strain enhancement ratio through machine learning. The study provided a reliable and robust methodology for the FRP composite, contributing to its design procedure. Pang Chen [10] evaluated the existing axial compressive constitutive model of FRP-confined circular concrete columns and established a predictive model for the axial compressive mechanical behaviors of FRP-confined concrete structures.

Many scholars have conducted studies on the use of FRP materials to enhance the blast resistance of concrete structures [11,12,13]. Xie Wei et al. [14,15] used FRP materials to strengthen underground straight-wall arch structures subjected to blast effects. Firstly, the straight wall arch was reinforced with internally applied FRP cloth, and through the TNT explosion test, it was found that the FRP cloth internal reinforcement method was effective in improving the blast resistance of the concrete arch structure, but the FRP-concrete interface peeling damage that appeared in the test showed that the simple internal FRP cloth reinforcement measure could not fully exploit the high strength and high-efficiency performance of the new composite material.

Zhao Chengjie et al. [5] systematically investigated the effect of FRP reinforcement on enhancing the blast resistance performance of concrete arches by conducting field model explosion tests. Different types of FRP reinforcement were applied to the concrete arch, including FRP pasted at different locations of the arch, the different number of FRP strips used, and increased restraint of FRP in the lateral direction for some of the arches. The effects of different FRP reinforcement methods, the effects of different FRP reinforcement rates, and the blast resistance of plain concrete arches after FRP reinforcement were analyzed through experimental studies. The results of the blast tests show that the FRP reinforcement can effectively improve the damage pattern of concrete arches under the blast action, but the FRP without additional restraint by only longitudinal FRP cloth paste will often fail to give full play to the reinforcement due to spalling. Therefore, the composite reinforcement method using longitudinal FRP paste and adding circumferential winding FRP for restraint can improve the blast resistance of the structure to the greatest extent, and the composite reinforcement method using longitudinal FRP paste and adding transverse U-shaped restraint has a better reinforcement effect.

In addition to enhancing the blast resistance of concrete arches, enhancing the static performance of arches by FRP materials is also a research focus. Zhang Xu et al. [16] carried out an experimental study of FRP strengthened reinforced concrete semicircular arch structure under quasi-static load, and the results showed that the cracking load of the structure was increased by 83.3~172% after using FRP reinforcement, and the structural stiffness and ductility were improved to a certain extent. Using the composite reinforcement method of FRP sheet with FRP cloth wrapped around it could increase the ultimate load of the structure by 15%. The average load carrying capacity can still maintain approximately 85% of the peak load after initial damage.

**Figure 2:**
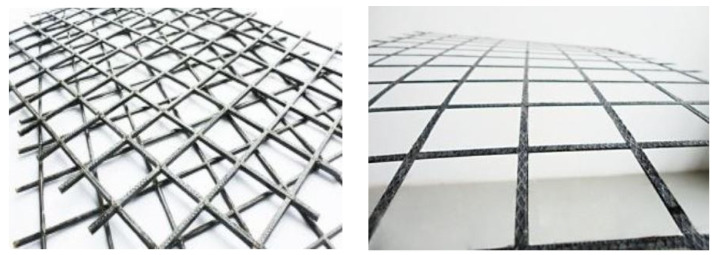
The FRP grid.

The FRP grid (Figure 2), as a newly developed new high-performance structural reinforcement material, is in the transition stage from laboratory research to engineering applications [17,18,19]. FRP lattices are made from continuous fiber yarns, which are formed longitudinally and horizontally into lattices by a special process and then dried and molded by infiltrating resin. Liu Yang et al. [20] used sprayed polyurea with FRP grid to reinforce concrete arch members, specifically including spraying only on the inner side of the arch, spraying on the whole surface of the arch, and spraying on the inner side of the arch with FRP grid. The damage patterns of cracking, spalling, and crushing of the concrete on the arch were found successively with the decrease of the proportional distance through the blast test, and the residual bearing capacity of the arch was tested using a quasi-static mid-span concentrated load test after the blast test to quantitatively assess the damage degree of the arch after the blast. The test results show that the sprayed polyurea coating has more excellent spalling resistance than the reinforcement method of pasting FRP and can greatly improve the blast resistance of concrete arches. The sprayed polyurea reinforcement on the full surface of the arch is the most effective. However, spraying polyurea reinforcement can hardly help to improve the static load-bearing performance of the arch structure.

Combined with previous research, in order to propose a more convenient, reliable, and cost-effective reinforcement method to improve the anti-explosion performance of the protective arch structures, the FRP grid and mortar composite reinforcement form is proposed, and the anti-explosion experiment of the composite arch was designed and conducted in the field. Considering the actual situation that reinforcement can only be carried out on the inner surface of the structure in the process of strengthening and renovation of the actual arch structure, in this paper, based on the previous study, the FRP grid is used instead of FRP cloth, and the ordinary mortar is used instead of polyurea in the inner surface strengthening of the arch structure. The feasibility of FRP grid and mortar composite reinforcement form is clarified through the experimental study of the blast resistance performance of FRP grid and mortar reinforced concrete arch.

## 2. Experiment Scheme

### 2.1. Composite Arch Structure

**Figure 3:**
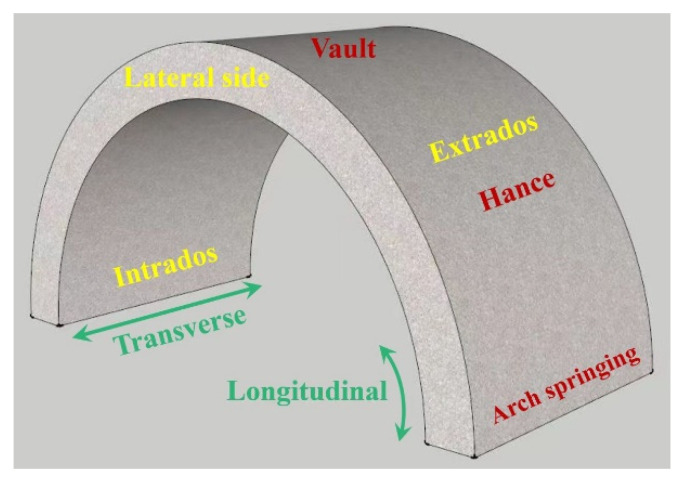
Concrete arch structure.

**Figure 4:**
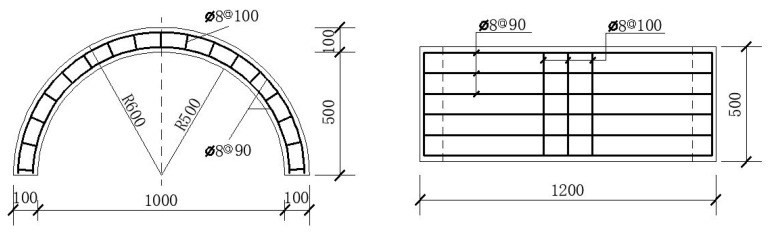
Reinforcement diagram of RC arch (mm).

The internal span of the arch is 1000 mm, the thickness is 100 mm, and the transverse width is 500 mm, as shown in Figure 3. The compressive strength of the concrete is approximately 29.1 MPa, and the tensile strength of concrete is 2.5 MPa. The reinforcement ratio of steel bars is about 1%. The elastic modulus of the steel bars is 200 GPa, the yield strength is 335 MPa, and the ultimate tensile strength is 455 MPa. The reinforcement diagram is shown in Figure 4 and the mechanical properties of concrete and steel bars are listed in Table 1. In Figure 3, the closed stirrups (Φ8@100) are located on the outside of the longitudinal reinforcements (Φ8@90) and have a restraining effect on them. Therefore, the thickness of the concrete cover shown in the Figure 4 is the distance from the edge of the concrete to the outer edge of the stirrups.

**Figure 5:**
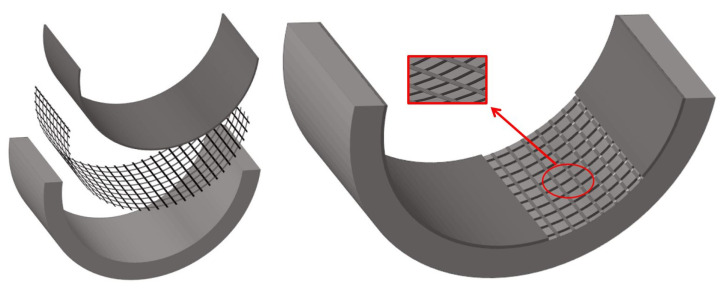
BFRP grid & mortar strengthened concrete arch structure.

Using basalt fiber-reinforced composite (BFRP) as the reinforcing material, Figure 5 shows the process of strengthening concrete arch structure with BFRP grid and mortar. After the natural curing of the structure, BFRP grid is firstly pasted and sand-bonded on the inner side of the arch, and half the number of grid intersections are anchored into the concrete by anchor nails, after which the BFRP grid reinforcement layer is smoothed with mortar, and the design thickness of mortar layer is 10 mm. In order to make the mortar layer better protect the concrete arch, the gradient design idea is used to design the mortar layer as a sacrificial layer, i.e., the strength of mortar is lower than the strength of concrete. The thickness of the BFRP grid frame is 1 mm, the grid size is 50 mm × 50 mm, and the material properties of the BFRP grid are listed in Table 2.

### 2.2. Anti-Explosion Experiment

**Figure 6:**
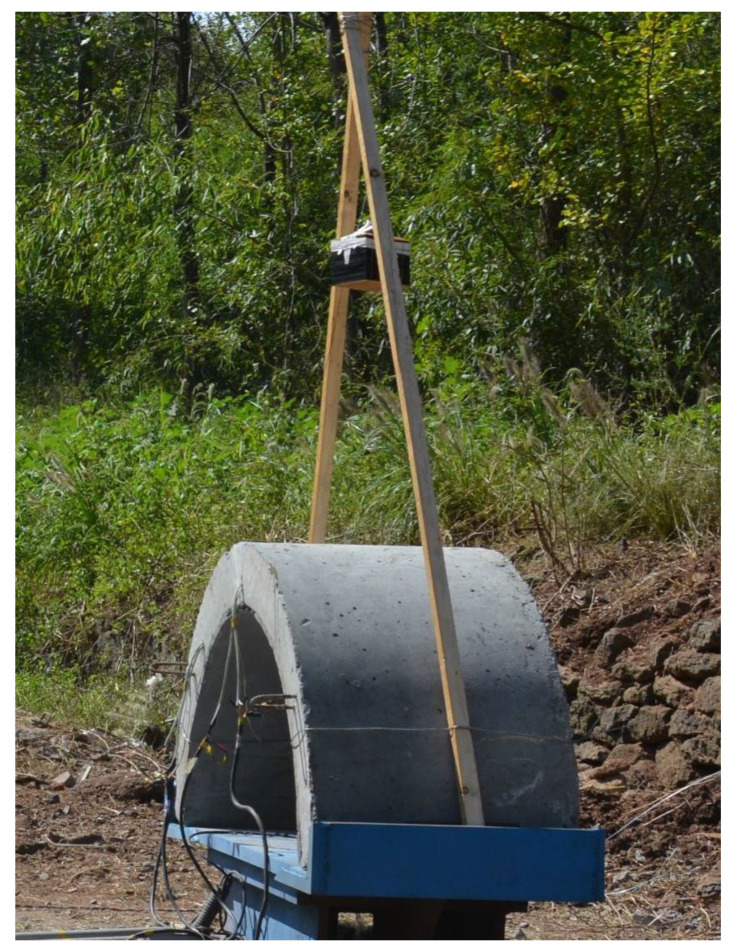
Anti-explosion experiment scheme of the arch structure.

In the experiment, the type of explosive used for air blast is TNT. As a type of chemical explosion, TNT explosion is an extremely rapid energy release process. In this instant, the energy contained in TNT is released at a very fast rate, and transformed into other forms of energy, such as kinetic energy, light, heat, etc. The shock wave generated by the explosion on the concrete elements of the effect is obvious. Air blast refers to a certain amount of equivalent TNT block placed at a certain distance from the top of the arch structure explosion, as shown in Figure 6. Displacement on both sides of the arch is restrained during the test to simulate simple support restraint.

As shown in Figure 6, the arch structure was placed on a stiff steel reaction frame and was impacted under TNT blasting. TNT block is placed at a certain height toward the upper surface of the arch. To restrain the opening of the arches at the springing, two steel plates were located at each end of the arch and were welded on the steel frame, so the restraint at the foot can be simplified into simply supported. The verticality of the specimens was verified before testing to avoid any instability effects. In this experiment, the TNT block is 2 kg, and the standoff distance is 600 mm.

## 3. Experiment Results

### 3.1. Arch Damage Mode

**Figure 7:**
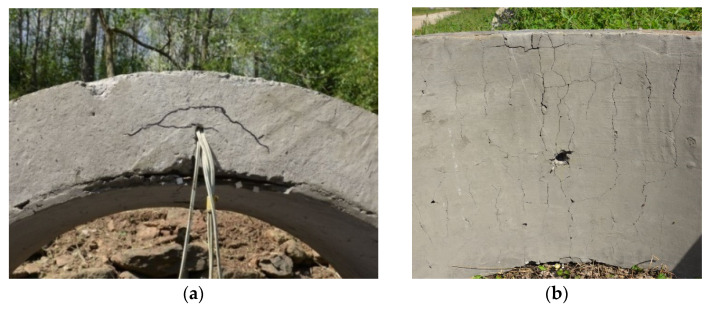
Damage of composite reinforced arch against blast.

After the explosion of 2 kg TNT at 600 mm from the upper surface of the arch, the FRP grid and mortar composite reinforced concrete arch structure showed plastic damage, and the damaged area was near the arch vault where it was closer to the explosive. A crack distributed basically along the axis of the arch, as shown in Figure 7a, indicates that the concrete at the arch vault showed a certain degree of spalling, but there was no falling of concrete. Since the FRP grid in the reinforcement layer is integrated with the arch structure through anchors, there is also no obvious spalling damage in the reinforcement layer.

From Figure 7b, it can be found that the mortar of the reinforcement layer shows more small cracks, which indicates that the reinforcement layer plays the role of flexural reinforcement and protects the integrity of the reinforced concrete arch through the deformation and energy absorption of the reinforcement layer. After the mortar layer cracked, the mortar layer did not fall off due to the restraining effect of the FRP grid.

### 3.2. Comparative Analysis of Arch Damages

**Figure 8:**
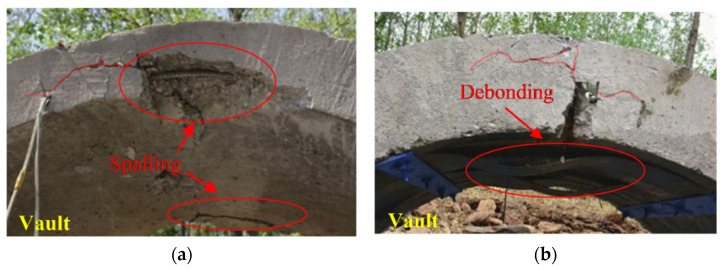
Anti-explosion damages of arches (**a**) A1, and (**b**) A4 [5].

In the literature [5], the anti-explosion conditions of Arch A1 and A4 are similar to those of the composite arch in this paper. Arch A1 has not been strengthened and serves as the control arch. Under the close-in explosion, A1 shows more severe spalling damage at the top of the arch, as shown in Figure 8a. Since Arch A1 is a reinforced concrete arch without CFRP reinforcement, bending cracks appeared at both hances, and concrete damage also occurred at the foot of the arch.

Arch A4 uses steel plate battens to anchor the bonded CFRP sheets. Due to the impact effect, the steel plate exactly fell off and the CFRP sheets debonded, the bolts were pulled out of the concrete, leaving a wide concrete crack, as shown in Figure 8b. Although the spalling damage of the arch was suppressed, the arch structure showed more serious vertical flexural cracks because the CFRP and steel sheets did not play a proper restraining role.

A comparison with arches A1 and A4 reveals that the concrete arches reinforced with FRP grid and mortar all have different degrees of improvement in damage patterns and improved blast resistance performance. Since the FRP grid is anchored to the concrete arch by anchor nails, the number of anchor nails is larger and the force is more uniform, which can provide better anti-blasting performance for the composite arch under dynamic load. The FRP grid and mortar reinforced arch can limit the appearance of large cracks in the arch structure by forming many small cracks in the reinforcement layer. This type of reinforcement can effectively inhibit the further expansion of concrete cracks, disperse large cracks into small ones, and effectively improve the overall working performance of the arch structure.

## 4. Conclusions

In this paper, the excellent material properties of FRP are successfully applied to the reinforcement and fabrication of the protective concrete arch structure. The reinforcement method of using the FRP grid & mortar at the bottom of the arch is proposed. The FRP grid and mortar reinforced concrete arch structure was designed and fabricated, and the blast resistance experiment was conducted in the field. The feasibility of FRP grid and mortar composite reinforcement form is clarified through the experimental study. It is concluded that:

- The blast resistance of FRP grid and mortar reinforced concrete arch was significantly higher than that of the unreinforced arch. The FRP grid and mortar composite reinforcement technology can be used to enhance the blast resistance of arch structures in protection projects.
- By analyzing the blast resistance test results and comparing them with the FRP and steel plate reinforced arch in the literature, the concrete arch reinforced with FRP grid and mortar has different degrees of improvement in damage patterns and improved blast resistance performance. The new composite grid and mortar reinforcement is useful for controlling crack expansion, inhibiting concrete spalling, and improving structural loading performance. This type of reinforcement can effectively inhibit the further expansion of concrete cracks, disperse large cracks into small ones, and effectively improve the overall working performance of the arch structure.
- From the viewpoint of maintaining the integrity of the blast-resistant structure, the new FRP grid and mortar reinforcement method is more advantageous than the traditional reinforcement method and can reduce the reinforcement cost while realizing the blast resistance effectiveness.

This paper only studies the reinforcement and anti-explosion performance of the semicircular arch structure. Since the mechanical properties of the arch will change with the change of the central angle, in the future, the reinforcement research of arch structures with different central angles can be carried out. In addition, the arch structure in this research is made of ordinary concrete. In order to reasonably match the mechanical properties of the material, the strength of the mortar used for reinforcement is not high. In the next step, ultra-high-performance mortar can be used to strengthen the high-strength concrete arch structure.

## Figures and Tables

**Table 1:** Mechanical properties of concrete and steel bars.

Concrete			Steel Bar		
Tensile Strength $\sigma_{tc\max}$	Compressive Strength $\sigma_{cc\max}$	Elastic Modulus $E_{c}$	Elastic Modulus $E_{s}$	Yield Strength $\sigma_{ts}$	Ultimate Strength
2.5 MPa	29.1 MPa	30 GPa	200 GPa	335 MPa	455 MPa

**Table 2:** Material properties of BFRP.

Thickness /mm	Fiber Area/(mm^2^)	Ultimate Load/N	Strength /MPa	Modulus of Elasticity/GPa	Fracture Strain
1	1.81	4013	2217	93.4	2.37%

## Data Availability

The data presented in this study are available on request from the corresponding author.

## References

[1] Yuan Y., Sun H., Chen Z., Bai E., Sun H. (2022). Numerical Simulation of Damping Performance of Elastically Supported Underground Arch Structures Subject to Penetration and Explosion. Adv. Civ. Eng..

[2] Simos N., Manos G.C., Kozikopoulos E. (2018). Near- and far-field earthquake damage study of the Konitsa stone arch bridge. Eng. Struct..

[3] Guangkun L., Wei W., Ruichao L., Weiming Z., Qiang Z. (2020). Deriving formulas of loading distribution on underground arch structure surface under close-in explosion. Eng. Fail. Anal..

[4] Flathau W.J., Sager R.A. (1964). The Design of Buried Arches to Resist Loads.

[5] Zhao C., Liu Y., Wang P., Jiang M., Zhou J., Kong X., Chen Y., Jin F. (2021). Wrapping and anchoring effects on CFRP strengthened reinforced concrete arches subjected to blast loads. Struct. Concr..

[6] Wang P., Chen H., Zhou J., Zhou Y., Wang B., Jiang M., Jin F., Fan H. (2018). Failure mechanisms of CFRP-wrapped protective concrete arches under static and blast loadings: Experimental research. Compos. Struct..

[7] Effiong J.U., Ede A.N. (2022). Experimental Investigation on the Strengthening of Reinforced Concrete Beams Using Externally Bonded and Near-Surface Mounted Natural Fibre Reinforced Polymer Composites—A Review. Materials.

[8] Tiwary A.K., Singh S., Kumar R., Sharma K., Chohan J.S., Sharma S., Singh J., Kumar J., Deifalla A.F. (2022). Comparative Study on the Behavior of Reinforced Concrete Beam Retrofitted with CFRP Strengthening Techniques. Polymers.

[9] Milad A., Hussein S.H., Khekan A.R., Rashid M., Al-Msari H., Tran T.H. (2022). Development of ensemble machine learning approaches for designing fiber-reinforced polymer composite strain prediction model. Eng. Comput..

[10] Chen P., Wang H., Cao S., Lv X. (2022). Prediction of Mechanical Behaviours of FRP-Confined Circular Concrete Columns Using Artificial Neural Network and Support Vector Regression: Modelling and Performance Evaluation. Materials.

[11] Hamed E., Chang Z.T., Rabinovitch O. (2015). Strengthening of Reinforced Concrete arches with Externally Bonded Composite Materials: Testing and Analysis. J. Compos. Constr..

[12] Ostad-Ali-Askari K.A.V.E.H., Singh V.P., Dalezios N.R., Crusberg T.C. (2018). Methods of strengthening reinforced concrete structures and introduction to the method of FRP sheet reinforcement. Methods.

[13] Anas S.M., Alam M. (2022). Performance of brick-filled reinforced concrete composite wall strengthened with CFRP laminate (s) under blast loading. Mater. Today Proc..

[14] Xie W., Jiang M., Chen H., Zhou J., Xu Y., Wang P., Fan H., Jin F. (2014). Experimental behaviors of CFRP cloth strengthened buried arch structure subjected to subsurface localized explosion. Compos. Struct..

[15] Chen H., Xie W., Jiang M., Wang P., Zhou J., Fan H., Zheng Q., Jin F. (2015). Blast-loaded behaviors of severely damaged buried arch repaired by anchored CFRP strips. Compos. Struct..

[16] Zhang X., Wang P., Jiang M., Fan H., Zhou J., Li W., Dong L., Chen H., Jin F. (2015). CFRP strengthening reinforced concrete arches: Strengthening methods and experimental studies. Compos. Struct..

[17] Guo R., Ren Y., Li M., Hu P., Du M., Zhang R. (2020). Experimental study on flexural shear strengthening effect on low-strength RC beams by using FRP grid and ECC. Eng. Struct..

[18] Zheng A., Liu Z., Li F., Li S. (2021). Experimental investigation of corrosion-damaged RC beams strengthened in flexure with FRP grid-reinforced ECC matrix composites. Eng. Struct..

[19] Sha X., Wang Z., Feng P., Yang J.-Q. (2020). Axial compressive behavior of square-section concrete columns transversely reinforced with FRP grids. J. Compos. Constr..

[20] Liu Y., Wang P., Jin F., He H., Zhou Y., Chen H., Zhou J., Wang B., Fan H. (2021). Blast responses of polyurea-coated concrete arches. Arch. Civ. Mech. Eng..

